# Different grades of green tea ‘Duyun Maojian’: a comprehensive constituent, bioactivity, and sensory evaluation from the consumer’s specific perspective

**DOI:** 10.1016/j.fochx.2026.103843

**Published:** 2026-04-07

**Authors:** Wei-wu Xia, Ting Li, Hui-min Fang, Fei-bi Xiong, Lu-lu Deng, Jiang Li, Xiao-jiang Hao, Peng Zhang, Shu-zhen Mu

**Affiliations:** a State Key Laboratory of Discovery and Utilization of Functional Components in Traditional Chinese Medicine, Natural Products Research Center of Guizhou Province, School of Pharmaceutical Sciences, Guizhou Medical University, Guiyang 550014, China; b College of Pharmacy, Guizhou University, South Section of Huaxi Road 2078, Guiyang 550025, China; c College of Pharmacy, Guizhou University of Traditional Chinese Medicine, Huaxi University Town, Guiyang 550025, China; d Kunming Institute of Botany, Chinese Academy of Sciences, 132 Lanhei Road, Kunming 650201, China

**Keywords:** Maojian, Sensory, Anti-inflammatory, Anti-thrombosis, Green tea

## Abstract

Duyun Maojian is a geographical-landmark product of Guizhou, with the processing technology included on the list of intangible cultural heritage. There are a few reports on the processing features of this green tea. This study investigated differences among five grades of Duyun Maojian by integrating objective component analysis with subjective aspects, including functional and sensory evaluations. The GC-MS analysis identified 78 key differential aroma components, primarily terpenes, alkanes, and esters, that contributed to the differences. LC-MS/MS data identified 23 key differential metabolites, including flavonoids and amino acids, as the primary differential metabolites for grade discrimination. Antioxidant, anti-inflammatory, and anti-thrombotic assays demonstrated the significant potential of Duyun Maojian for vascular protection. Sensory evaluation revealed grade-dependent variations in bitterness and umami that align with trends in TPC and TFAA. This study indicated that picking timing, picking part, and processing applicability play vital roles in determining tea quality, and smell is an essential basis for grading.

## Highlights

• The difference of five grades of Duyun Maojian was determined by GC-MS and LC-MS/MS.
• Human olfactory is more important than instrument detection for grade classification.
• Lower-grade Duyun Maojian showed more prominent chestnut and woody aromas than higher grades.
• Duyun Maojian protects the vascular through anti-inflammatory and anti-thrombotic effects.

## 1 Introduction

Green tea, the most widely consumed beverage after water worldwide, has gained widespread popularity for its distinctive flavor and numerous health benefits (Khan & Mukhtar, 2018). These benefits of green tea include antioxidant properties, anti-obesity effects, regulation of lipid metabolism, vascular protection, and neuroprotection, among others (Kochman et al., 2020). The cardioprotective role of green tea catechins has been further highlighted by recent clinical and mechanistic studies (Eshraghi et al., 2025). Owing to substantial variations in cultivation conditions and processing techniques for green tea, the interplay among quality development, flavor profile, and functionality across diverse high-quality teas has consistently attracted scholarly attention.

Duyun Maojian, recognized as a national geographic indication product, is cultivated on Yunwu Mountain, situated at the confluence of Duyun City and Guiding County in Guizhou Province. A unique historical trajectory marks the evolution of the Maojian brand. Notably, in 1956, Chairman Mao Zedong bestowed the name 'Maojian' on the tea (Qian, 2009). By 2024, the cultivation area had expanded to more than 1.2 million acres in Qiannan Prefecture, and the brand's value had reached 4.89 billion yuan (Xinhua-AsiaNet., 2024). Duyun Maojian continues to be acknowledged as one of the ten most renowned teas in China.

The processing of Duyun Maojian tea differs from that of conventional green tea. According to local standards (DB52/T 433-2018), fresh leaves (one bud or one bud and one leaf, < 2 cm) are handpicked from early March to May. Post-harvest, the leaves undergo spreading, fixation, rolling, ball rubbing, pekoe emergence, and drying to produce the final products. A complex craft that imparts the tea's characteristic curled shape and white pekoe. In 2022, Duyun Maojian's production techniques were recognized as a representative example of humanity's intangible cultural heritage (China-Daily, 2022). In a recent study, Liao et al. conducted a systematic investigation of the dynamic alterations in non-volatile metabolites during the processing of Duyun Maojian tea, elucidating the mechanisms by which these changes influence the development of taste quality (X. Zhou et al., 2026). In the commercial market, Duyun Maojian is available in five grades: 'Supreme' (zunpin), 'Premium' (zhenpin), 'Special' (teji), 'Grade I' (yiji), and 'Grade II' (erji), with quality differences among them potentially being a primary consideration for consumers. The substantial variation in consumer sensory experiences plays a critical role in shaping purchasing decisions. The quality characteristics of five grades and consumers' awareness of this famous tea remain to be elucidated.

This study integrates chemical analysis, bioactivity assessment, and consumer-based sensory evaluation to address this research gap. The constituents of tea play a crucial role in defining its flavor and health-promoting properties. The non-volatile constituents of tea include catechins, flavonoids and their glycosides, amino acids, low-molecular-weight phenolic acids, and alkaloids, among other compounds, and contribute to taste and functionality (Li et al., 2024). Consequently, this study conducted a semi-quantitative analysis of total polyphenols (TP), total flavonoids (TF), and total free amino acids (TFAA) and demonstrated their roles in consumer sensory attributes. Liquid chromatography-mass spectrometry (LC-MS) was used for differential component analysis to objectively assess the status of Maojian grades. The volatile compounds are primarily responsible for tea's aroma, with the key aromatic components being olefins, esters, and alcohols (Tang et al., 2025; F. Wang et al., 2025). Therefore, gas chromatography-mass spectrometry (GC-MS) was employed to analyze aroma compounds and compare the results with the subjects' sensory results. Additionally, this study examined the vascular-protective properties of the five grades of Dunyun Maojian by evaluating antioxidant, anti-inflammatory, and antithrombotic activities, as these features may attract the attention of ordinary consumers, especially for those with high-sugar, high-fat dietary intake in their daily lives. The integrated analysis of differential-grade compounds, flavor components, consumer sensory attributes, and vascular-protective effects will enhance understanding of the quality characteristics of this distinctive food from Southwestern China and highlight its potential role in human health.

## 2 Materials and methods

### 2.1 Sample and reagents

Five commercial grades of Duyun Maojian ('Supreme', 'Premium', 'Special', 'Grade I', and 'Grade II') were obtained directly from Guizhou Qinxin Xiaozhu Tea Co., Ltd. (Duyun, China). All grades were produced in the same harvest season (spring 2025) from a single tea plantation (Yunwu Mountain) to minimize environmental variability. Grade classification followed the provincial standard DB52/T 433-2018, primarily based on leaf tenderness and picking time. Samples (500 g per bag) were purchased and stored at -20 °C until analysis.

L-Theanine (HPLC ≥ 98%), gallic acid (HPLC ≥ 98%), and Ninhydrin (HPLC ≥ 95%) were purchased from Shanghai Yuanye Bio-Technology Co., Ltd. (Shanghai, China). Rutin (HPLC ≥ 98%) was purchased from Baoji Herbest Bio-Tech Co., Ltd (Baoji, China). Folin & Ciocalteu’s Phenol Reagent was provided by Beyotime Biotechnology Co., Ltd (Shanghai, China). The DPPH Free Radical Scavenging Capacity Assay Kit and the ABTS Free Radical Scavenging Capacity Assay Kit were purchased from Beijing Solarbio Science & Technology Co., Ltd (Beijing, China).

DMEM medium was purchased from the Thermo Fisher Scientific Co., Ltd (Waltham, U.S.A.). Fetal bovine serum was sourced from Biological Industries (Kibbutz Beit, Israel). The penicillin-streptomycin-amphotericin B mixture was obtained from Wuhan Pricella Biotechnology Co., Ltd (Wuhan, China). RAW 264.7 cells were purchased from the Cell Bank of the Chinese Academy of Sciences. (Shanghai, China). Lipopolysaccharide (LPS) was purchased from Sigma-Aldrich (Shanghai) Trading Co., Ltd (Shanghai, China), and ammonium pyrrolidinedithiocarbamate (PDTC) was sourced from Beyotime Biotechnology Co., Ltd (Shanghai, China). AB series wild-type zebrafish were purchased from Shanghai FishBio Co., Ltd (Shanghai, China). (±)-Epinephrine hydrochloride (HPLC ≥ 98%) was purchased from Shanghai Aladdin Biochemical Technology Co., Ltd (Shanghai, China). Aspirin (Acetylsalicylic acid, HPLC ≥ 99%), *o*-dianisidine (3, 3'-dimethoxyebenzidine, HPLC ≥ 97%) was sourced from Shanghai Macklin Biochemical Technology Co., Ltd (Shanghai, China).

The HPLC-grade methanol, acetonitrile, and formic acid were supplied by Energy Chemical (Shanghai, China). Pure water was sourced from Wahaha Co., Ltd (Hangzhou, China). Anhydrous ethanol and other analytical-grade reagents were obtained from Guizhou Excellence Sheng Technology Co., Ltd (Guiyang, China).

### 2.2 Total polyphenol, total flavonoid, and total free amino acids contents

Different grades of Duyun Maojian tea samples (100 g) were ground and weighed 100 mg precisely in triplicate, and then transferred into test tubes. Next, 10 mL of 70% methanol/water (v/v) was added at a solid-liquid ratio of 1:100 (g/mL) and heated in a water bath (70 °C) for 20 min. After cooling to room temperature, 4 mL of the supernatant was transferred to 15 mL centrifuge tubes and centrifuged at 4000 rpm for 15 min. The supernatant (1 mL) was then pipetted into a 15 mL centrifuge tube and diluted 10-fold with 70 % methanol/water (v/v) for the test.

The total polyphenol content (TPC) was determined by the Folin-Ciocalteu method (Y.-Y. Zhang et al., 2023). Folin-Ciocalteu reagent (1.5 mL, 0.25 mol/L) and the sample test solution (100 μL) were mixed and reacted for 5 min. Subsequently, 1.5 mL of 20% (w/v) Na_2_CO_3_ was added, and the reaction was carried out at room temperature for 60 min. Finally, the absorbance was measured at 760 nm using an ultraviolet spectrophotometer. The standard curve (R^2^ = 0.9982) was plotted using gallic acid standard solutions (40, 60, 80, 120, 160, and 200 μg/mL) instead of the test solution. TPC was expressed as μg of gallic acid equivalents (GAE)/g of dry mass (DM) of the tea sample.

The total flavonoid content (TFC) was measured according to the reported method, with slight modifications (L. Wang et al., 2018). Briefly, 0.2 mL of the tea sample test solution was mixed with 0.3 mL of 70% methanol/water (v/v). Afterward, 30 μL of NaNO_2_ solution (5%, w/v) was added, mixed, and kept at room temperature for 5min, followed by the addition of 30 μL of AlCl_3_ (10%, w/v). After the reaction for 6 min, 0.2 mL of NaOH (1 mol/L) was added, and the final volume was adjusted to 1 mL with 70% methanol/water. This solution was vortexed completely and incubated at 35 °C for 30 min. 200 μL of each sample was pipetted into a 96-well plate, and the absorbance was measured at 510 nm using a microplate reader (SpectraMax M2, Molecular Devices, USA). The standard curve (R^2^ = 0.9957) was plotted by using rutin standard solutions (40, 60, 100, and 140, 160 μg/mL) instead of the test solution as described, and the TFC was expressed as μg rutin equivalents (RE)/g dry mass (DM) of tea samples.

The total free amino acid (TFAA) content was determined using the method described by Jabeen et al., with some modifications (Jabeen et al., 2019). In brief, 400 μL of the tea sample test solution was mixed with 200 μL of phosphate buffer (0.1 mol/L, pH 8.0) and 400 μL of the ninhydrin solution (2%). The mixture was heated in a metal bath at 100 °C for 13 min. After cooling to room temperature, the absorbance was measured at 570 nm using a microplate reader. _L_-Theanine (100, 200, 300, 400, 500 μg/mL) was used as a substitute for the sample solution in the reaction, and a 10-fold diluted solution was used to establish a standard curve (R^2^=0.9936). The TFAA was expressed as μg _L_-theanine equivalents (TE)/g of tea sample (dry mass).

All samples were tested in triplicate. All reported values were expressed as mean ± standard deviation (SD).

### 2.3 Flavor components analysis by GC–MS

The volatile compounds in the Duyun Maojian samples were extracted using headspace solid-phase microextraction (HS-SPME) with a 50/30 μm DVB/CAR/PDMS fiber (Supelco, USA). Tea samples (1.0 g) were placed in headspace vials with 6 mL of boiling water and heated to 70 °C for 10 min. For each sample, 200 μg ethyl decanoate was added as an internal standard for signal correction. Before extraction, the SPME fiber was conditioned through thermal desorption in the GC injection port at 27 °C. Eventually, the sample was manually injected after a 40 min of extraction.

The aroma profiles of the tea samples were analyzed by GC-MS (Agilent 8890-7000D, Agilent Technologies, Santa Clara, CA, USA) equipped with an HP-5ms Ultra Inert capillary column (30 m × 0.25 mm × 0.25 μm; Agilent 19091S-433UI). High-purity helium was employed as the carrier gas at a constant flow rate of 1 mL/min. The mass spectrometer was operated in electron ionization (EI) mode at 70 eV, with the ion source temperature set to 230 °C. Other relevant configuration parameters are listed in Section 1 of the Supplemental Information (SI).

The GC-MS data were preprocessed using the GNPS (http://gnps.ucsd.edu) GC-MS EI Data Analysis workflow. The compounds were identified automatically by the Spectral Library Search. The quantitative tables from the workflow on the GNPS platform were preprocessed using the MetaboAnalyst online platform (version 6.0, https://www.metaboanalyst.ca/) (Pang et al., 2024).

### 2.4 Non-volatile compounds analysis by LC-MS

The samples were pulverized and accurately weighed at 100 mg in triplicate. Each 100 mg sample was extracted with 4 mL of 70% methanol/water (v/v) and sonicated for 30 min at room temperature. The extracts were diluted 2.5-fold with 70% methanol/water (v/v) and centrifuged at 12,000 rpm for 10 min. The obtained supernatant was filtered through a 0.22 μm membrane for LC-MS injection. Quality control (QC, a mixture of equal volumes of each test solution) and blank solution (70% methanol/water) were prepared simultaneously for MS response signal correction and background subtraction.

An ultra-performance liquid chromatography (UPLC) system (Ultimate 3000, Dionex, Sunnyvale, CA, USA) coupled to a mass spectrometer (Q-Exactive Focus, Thermo Fisher Scientific, Waltham, MA, USA) with an electrospray ionization (ESI) source was used for analysis. Compounds were separated using an ACQUITY UPLC HSS T3 column (1.8 μm, 2.1 mm (inside diameter) × 100 mm (length), Waters Corp., Milford, MA, U.S.A) at a flow rate of 0.2 mL/min (mobile phases A, 0.1 % formic acid in water; mobile phases B, 0.1 % formic acid in acetonitrile). The injection volume was 2 μL, and the column temperature was maintained at 30 °C. The linear elution gradient was set as follows: 0-1 min, 10% B; 1-2.5 min, 10-15% B; 2.5-7 min, 15-18% B; 7-8 min, 18-10% B; 8-10 min, 10-16% B; 10-10.5 min, 16-18% B; 10.5-12 min, 18-22% B; 12- 14 min, 22-23% B; 14-15 min, 23-95% B; 15-18 min, 95% B; 18-19 min, 95-10% B; and 19-21 min, 10% B. The acquisition was conducted in the positive mode with a spray voltage of 3400 V and a capillary temperature of 350 °C. Sheath and auxiliary gas were set to 45 and 15 arbitrary units, respectively. The MS scanning mode was configured to full MS data-dependent MS^2^ (dd-MS^2^) mode, with a resolution of 70,000 and a scan range of 100–1500 (*m/z*) for MS^1^, and a resolution of 17,500 for MS^2^. The high-energy collision dissociation was configured in a stepped mode, with the energy levels set to 30, 40, and 60 eV. The other detailed LC-MS parameters are available in Section 2 of the SI.

All raw LC-MS data (.raw format) were converted to '.mzML' format by MSConvert with filtering parameters set to MS levels 1-2 and peak picking. The MZmine software (version 4.3.0; detailed parameter settings are provided in Section 3 of the SI) was used for mass detection, chromatographic peak building, deconvolution, isotope filtering, feature alignment, and feature list row filtering (Schmid et al., 2023). The MS^1^ feature quantification table was further filtered and normalized using QC samples in RStudio (R code in Section 4 of the SI). The feature-based molecular network was created by uploading the .mgf file of the MS^1^ feature quantification table to the GNPS platform (https://gnps.ucsd.edu/). The compounds were annotated using GNPS multi-library matching and a self-constructed library search, with a metabolomics identification level 2.

The relevant parameters were set as follows: (1) basic options: precursor ion mass tolerance and fragment ion mass tolerance, 0.02Da; (2) advanced network options: min pairs cos: 0.7, network topk: 10, minimum matched fragment ions: 6, maximum shift between precursors: 500 Da; (3) other advanced options being set by default (M. Wang et al., 2016).

### 2.5 Cardiovascular system protective effect

The vascular system-protective activity was evaluated using antioxidant, anti-inflammatory, and anti-thrombotic assays. The pulverized sample (10.0 g) was ultrasonically extracted with 100 mL of 70% ethanol/water for one hour. The extraction was repeated three times. Finally, the obtained filtrates were combined and concentrated for testing.

#### 2.5.1 Antioxidant activity

The free radical scavenging ability was tested through the DPPH and ABTS assay kit. The experimental protocol followed the manufacturer's guidelines (Teixeira Oliveira et al., 2023; Wu et al., 2023). For the DPPH assay, 10 μL of the sample solution and 90 μL of the DPPH working solution were mixed at room temperature, away from light, for 30 min. In the blank group, the DPPH working solution was replaced with anhydrous methanol, whereas in the control group, the sample solution was replaced with anhydrous methanol. The absorbance was measured at 517 nm using a microplate reader (SpectraMax M2, Molecular Devices, USA). For the ABTS assay, 50 μL of the sample test solution and 150 μL of ABTS working solution were incubated at room temperature for 10 min, shielded from light. The blank group was set to 150 μL of anhydrous methanol instead of the ABTS working solution. The control group uses 50 μL of anhydrous methanol instead of the sample solution. The absorbance was determined at 734 nm. Vitamin C was used as a positive control in the DPPH and ABTS assays. The free radical scavenging ability was calculated through the formula: Scavenging rate (%) =[1-(A1-A2)/A3] ×100%, where A1, A2, and A3 are the absorbance of the experimental group, blank group, and control group.

#### 2.5.2 Anti-inflammation activity

The anti-inflammatory properties of the tea samples were assessed by quantifying the nitric oxide levels in an LPS-induced inflammation model using RAW264.7 cells. The RAW 264.7 mouse macrophage cell line was cultured in DMEM medium containing 10% fetal bovine serum (FBS) and 1% penicillin-streptomycin-amphotericin B mixture at 37 °C with 5% CO₂. Logarithmic-growth-phase cells were selected for the experiment.

For cell cytotoxicity assays, RAW 264.7 cells were inoculated into 24-well plates at 2×10 ^5 Cells/well and incubated for 12 h to allow adhesion. Fresh medium containing different concentrations of the tested samples (200, 100, 50 μg/mL) was used to replace the old medium in 24-well plates. After 24 h of incubation, 50 μL of CCK-8 reagent was added to each well, and incubation was continued for 2 h. The blank group contains only culture medium, and a control group using DMSO (≤ 0.1%, v/v) replaces the samples. The absorbance was measured at 450 nm using a microplate reader (SpectraMax M2, Molecular Devices, USA). The cell viability was calculated using the formula: Cell Viability (%) =[(As-Ab)/(Ac-Ab)] ×100%, where As, Ac, and Ab are the absorbance of the experimental, blank, and control groups.

For NO generation inhibition assays, RAW 264.7 cells were inoculated into 24-well plates and incubated for 12 h. After that, the old medium was replaced with fresh medium containing the test samples at different concentrations (400, 200, 100, and 50 μg/mL). After a 2 h incubation, 10 μL of LPS solution (0.25 mg/mL) was added to induce cellular inflammation. After a final 12 h incubation, 50 μL of the supernatant was transferred to a 96-well plate for nitric oxide detection using the Nitric Oxide Assay Kit (Beyotime) according to the manufacturer's instructions. The model group was without sample processing, and PDTC (40, 20, 10, 5 μM) was used as a positive drug. The absorbance was measured at 540 nm. NO generation inhibition rate was calculated through the formula: NO Inhibition (%) =[(C_m_-C_s_)/C_m_] ×100%, where C_m_ and C_s_ are the absorbance of the model and experimental group, respectively. The experiments were repeated three times for each group, and the results were expressed as mean ± SD.

#### 2.5.3 Anti-thrombotic activity

Duyun Maojian tea’s anti-thrombotic activity was evaluated using zebrafish thrombus models induced by (±)-Epinephrine hydrochloride (AH). Zebrafish (AB strains) were fed at 28.5 ± 1 °C with a 14 h/10 h light/dark cycle. The adult zebrafish were selected for breeding in a 2:1 ratio (male: female). The embryos were collected and cleaned, and transferred to petri dishes containing embryo medium. After cultivation for 3-6 h, embryos were sorted and disinfected using embryo medium containing 0.003% sodium hypochlorite. After cleaning, well-developed embryos were cultured at 28.5 ± 1 °C for further experiments. The Animal Care and Welfare Committee of Guizhou Medical University approved all zebrafish assays (No. 2502559).

The 72 hpf (hours post fertilization) embryos were randomly placed in a 24-well cell culture plate, with 15 larvae per well (three replicate wells per sample). The experimental group was treated with 15 μM AH and 40 or 20 μg/mL of the samples. The model group is adding 15 μM of AH and uses 0.1% DMSO to replace the sample solution. Aspirin (15 μg/mL) was used as a positive drug, and the blank group was without adding AH and samples. After administration at 28.5 ± 1 °C for 16 h, the zebrafish were anesthetized using tricaine (0.3%, m/v) and stained with *o*-dianisidine (0.6 mg/mL) for 30 min. Photographs of the caudal vein thrombosis in zebrafish larvae and the cardiac staining area were taken under a microscope (Olympus, SZX-16, Japan). Image J 1.5.4 software was used to process the images. All zebrafish experiments were approved by the Animal Care and Welfare Committee of Guizhou Medical University (No. 2502559) and were conducted in accordance with relevant national regulations and internationally recognized guidelines, including the NIH Guide for the Care and Use of Laboratory Animals.

### 2.6 Sensory evaluation

Tea sensory review methods followed the Chinese standard (GB/T 23776-2018). The sensory evaluation panel comprised 12 members with normal visual, gustatory, olfactory, and tactile functions (6 males and 6 females, ages from 24 to 30). The participants who received foundational training in review terminology, derived from the 'Sensory flavor wheel' published by the China Tea Research Institute. Before evaluation, the assessors were fully informed of the study requirements and risks and were required to ensure that they had not smoked, consumed alcohol, or eaten spicy or other irritant foods. Samples (labeled with random letters) were presented in a randomized and balanced order to minimize carry-over effects. The group members rinsed their mouths with distilled water between samples. For statistical analysis, significant differences in attribute scores among grades were determined using the non-parametric Kruskal-Wallis test followed by Dunn's post-hoc test (P < 0.05).

Briefly, each 2 g of tea sample was weighed and transferred to a glass, then 100mL of boiling water was added to brew the tea for 5 min (tea-water ratio, 1:50). The appearance, color, and aroma of the brewed tea were documented for the dried tea leaves. The color, smell, and taste of the tea infusion were assessed. The appearance of brewed tea leaves was described qualitatively. The taste-related attributes, namely bitterness, astringency, sweetness, umami, and intensity, were evaluated using a five-point scale, where 0 represented the lowest intensity, 2.5 indicated medium intensity, and 5 denoted the highest intensity.

### 2.7 Statistical analysis

All samples were analyzed in triplicate, and the results were expressed as mean ± SD. The multivariate statistical analyses, including principal component analysis (PCA) and partial least squares discriminant analysis (PLS-DA), were performed using SIMCA 14.1 (Umetrics AB, Umea, Sweden) to screen for differential features. Significant differences were analyzed using one-way ANOVA with P < 0.05 in Prism software.

## 3 Results and discussion

### 3.1 Total polyphenol, total flavonoid, and free amino acid contents

**Fig. 1:**
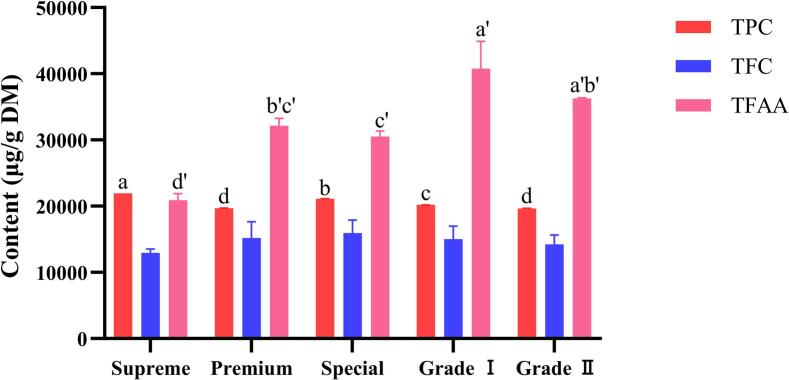
Contents of total polyphenol (TPC), total flavonoid (TFC), and total free amino acids (TFAA) for five different grades of Duyun Maojian. Values with the same superscript letters indicate no significant differences, whereas different superscript letters denote significant differences (P < 0.05).

Polyphenols, flavonoids, and free amino acids are the predominant constituents that affect the sensory quality and functional value of tea. The TPC, TFC, and TFAA were determined by the external standard method. Statistical analysis was performed through one-way analysis of variance (P < 0.05), with letters indicating significant differences. The results are shown in Fig. 1. TPC generally decreased with descending tea grade, except for 'Premium' tea. 'Supreme' tea exhibited the highest TPC (21936.86 μg/g), significantly differing from the other four grades (P < 0.05), while Grade II showed the lowest content (19662.95 μg/g), with no significant difference from 'Premium'. TFAA content was lowest in 'Supreme' (20884.85 μg/g), significantly differing from all other grades (P < 0.05). 'Grade I' (40775.76 μg/g) and 'Grade II' (36263.64 μg/g) exhibited the highest TFAA levels with no significant difference between them, both significantly higher than that of 'Premium' (32139.39 μg/g) and 'Special' (30524.24 μg/g). This pattern contradicts the conventional understanding of the "higher tenderness, higher amino acids". According to Duyun Maojian harvesting standards, 'Supreme' comprises unexpanded single buds; 'Premium' and 'Special' consist of one bud with one leaf; and Grade I and Grade II contain one bud with two leaves. Tenderness decreases progressively, and the growth period increases. Considering the materials' applicability to the process, specific parameters should be adjusted to meet the requirements of the rolling, ball rubbing, and pekoe emergence stages. We propose that differential amino acid accumulation may result from: (1) harvesting time variations affecting amino acid enrichment periods; (2) underdeveloped chloroplasts in unexpanded 'Supreme' buds leading to weaker photosynthesis and reduced amino acid synthesis (Gao et al., 2025; Xie et al., 2021); (3) fully expanded leaves in Grade I exhibiting mature chloroplasts with enhanced photosynthetic capacity, maximizing amino acid accumulation; and (4) processing parameters, particularly fixing temperature and rolling pressure, influencing amino acid transformation via Maillard reactions and protein hydrolysis (Aaqil et al., 2023; Lin et al., 2020). Theanine acid was observed to undergo conversion and coupling with catechins during tea processing, thereby reducing TFAA (P. Zhang et al., 2021). TFC content showed no significant differences across grades (12923.33-15950 mg/kg) (P > 0.05). This phenomenon likely reflects the multifactorial regulation of flavonoid accumulation, influenced by acquisition time, light exposure, and processing parameters, and reactions during drying (Ma et al., 2023; J. Zhou et al., 2025).

In conclusion, each grade of Duyun Maojian tea exhibits distinct chemical profiles: 'Supreme' is characterized by high TPC; 'Special' shows balanced TPC and TFC; and Grades I and II are enriched in TFAA. These variations arise from the combined effects of harvesting standards (tenderness and leaf/bud composition), harvesting time, and processing parameters. Such chemical differentiation provides a scientific basis for tea grading.

### 3.2 Volatile compounds analysis

**Fig. 2:**
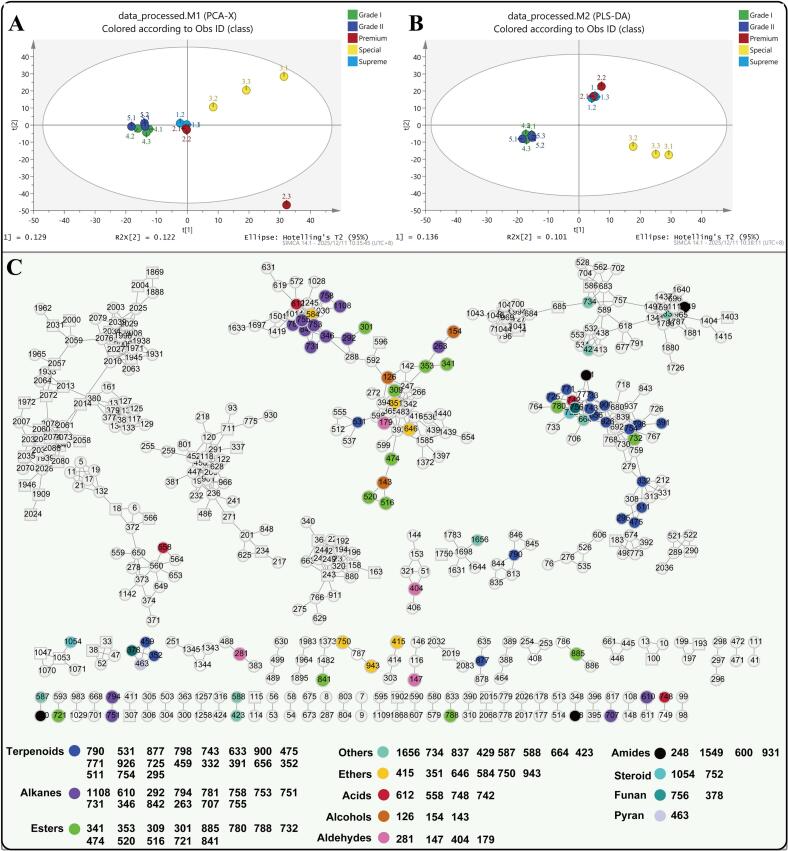
Volatile compounds analysis of five different grades of Duyun Maojian. (A) Principal component analysis plot of volatile components of Duyun Maojian. (B) PLS-DA plot of the defined three sample groups. (C) Molecular network classification annotation diagram based on GC-MS data.

**Fig. 3:**
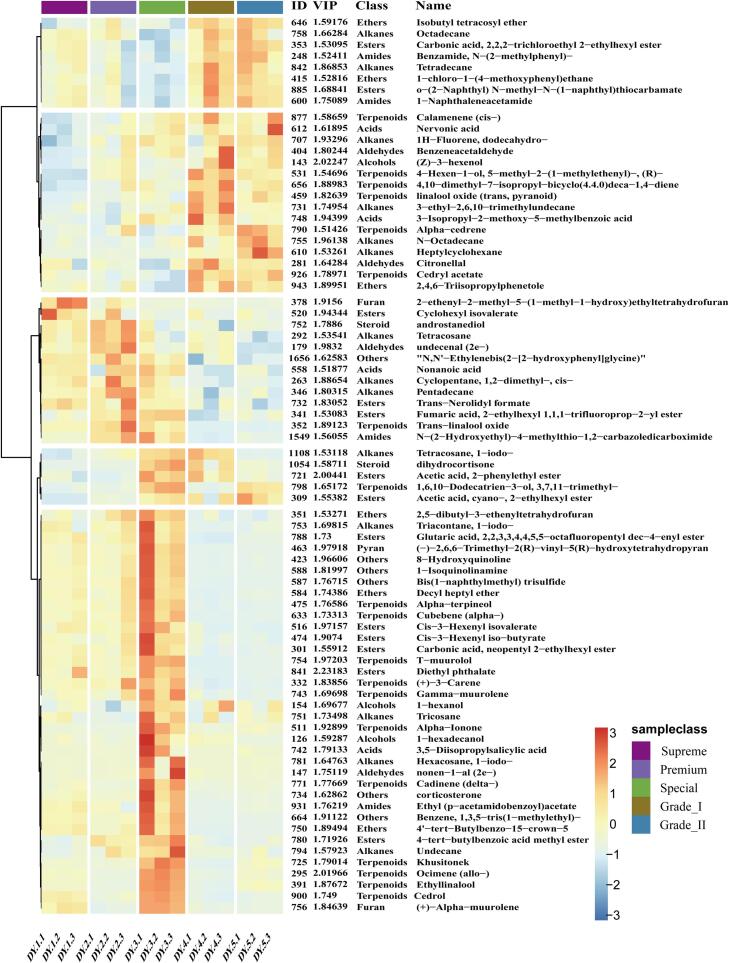
Relative abundance of intergroup differential volatile compounds in each sample.

GC-MS analysis was used to identify differential aroma components across different grades of Duyun Maojian. The multivariate statistical analysis of GC-MS data, combined with molecular networks, was used to better illuminate the differential aroma compounds in the five Maojian teas. After GC-MS EI workflow processing, 2088 compound features were detected, of which 284 were identified as aroma compounds. Following data filtering and imputation, a PCA model (R^2^X = 0.252) was constructed using unit-variance (UV)-scaling compound features. The results suggest a high degree of similarity in the aroma profiles between the 'Supreme' and 'Premium' categories, as well as between the 'Grade I' and 'Grade II' categories (Fig. 2A). The results implied that the aromatic components show a stepwise change as the tea grade decreases. To clarify the differential components between 'higher' ('Supreme', 'premium'), 'medium'('Special'), and 'lower' ('Grade I' and 'Grade II') grades, the PLS-DA model (R^2^X=0.237, R^2^Y=0.979, Q^2^=0.572, Fig. 2B) was established. 164 differential metabolites were confirmed using screening thresholds of VIP > 1.5, of which 78 differential aroma components were identified and labelled by different colors in the molecular network (Fig. 2C). The results indicated that terpenoids, alkanes, and esters contributed much more to the difference between the grades compared with other classes. Relative abundance of the identified differential compounds in five grades of Maojian tea was plot on Fig. 3. Terpenoids, such as 'calamenene(cis)', '4-Hexen-1-ol, 5-methyl-2-(1-methylethenyl)-, (R)-', '4,10-dimethyl-7-isopropyl-bicyclo (4.4.0) deca-1,4-diene', 'linalool oxide (trans, pyranoid)', '*α*-Terpineol', 'Cedryl acetate' are relative enriched in 'lower' ('Grade I' and 'Grade II') grades of teas. In 'Special' tea, the differential mark compounds display a wide variety of aroma categories, including ethers, esters, terpenoids, alkanes, amides, acids, aldehydes, pyran, furan, and alcohols, and others (Fig. 3). The differential aroma compounds among the three defined tea groups suggest that, during the continuous growth phase of fresh leaves, chemical substances undergo temporal transformations and exhibit spatial distribution. Therefore, the timing of harvest and the specific locations of buds and leaves are critical factors that directly influence tea volatile aroma.

### 3.3 Non-volatile compounds analysis

**Fig. 4:**
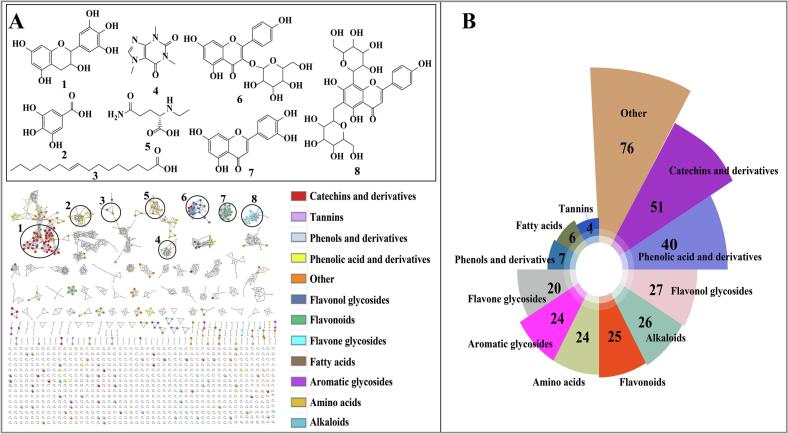
Feature-based molecular network (FBMN) of metabolites in Duyun Maojian detected by the UHPLC-Q-E (Orbitrap)-MS/MS. (A) Representative compounds in the molecular network diagram. The colored nodes indicate the distinguishing features of the components. Each node represents a feature of FBMN. The edges between the two connected nodes denote the cosine score, namely, the clustering similarity between the components. (B) Component classification diagram.

Non-volatile compounds also determine the quality of tea. The LC-MS/MS data were subjected to Mzmine data preprocessing steps to obtain 920 compound features, of which 330 components were identified from MS^2^ spectra and roughly categorized into 12 groups (Fig. 4A, 4B). Flavonoids, phenolic acids, and amino acids are the main categories, of which flavonoids are dominated by catechins, flavonoids and their derived structural types, flavone glycosides, and flavonol glycosides. This comprehensive metabolite profiling identification establishes a crucial foundation for quality-related biomarker investigations.

**Fig. 5:**
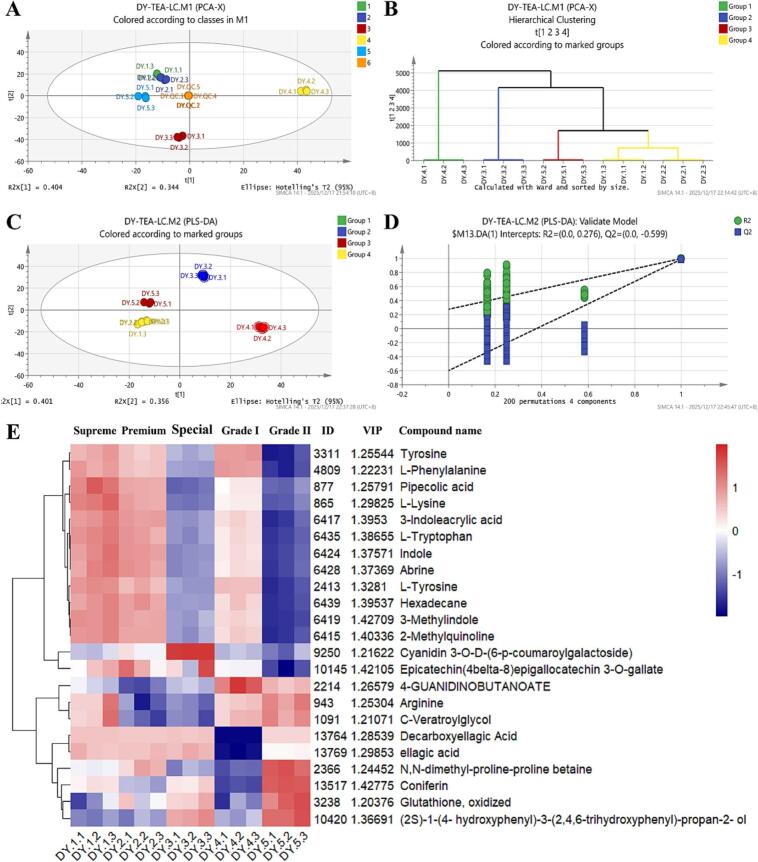
Results of differential metabolite screening based on LC-MS/MS data. (A) Principal component analysis; (B) Hierarchical cluster analysis (HCA); (C) Partial Least Squares Discrimination Analysis (PLS-DA); (D) Permutation test of PLS-DA model. (E) The relative abundances of the screened differential metabolites across different groups by VIP > 1.2 threshold based on PLS-DA model.

Multiple statistical analyses of the MS^1^ feature table were employed to reveal differences among components of Duyun Maojian grades. As shown in the PCA plot (Fig. 5A), the QC samples were tightly clustered in the center region, indicating good experimental reproducibility and data quality. The first two principal components accounted for 74.8% of the data's variation, capturing the primary direction of variation. The five tea grades showed distinct separation patterns in the principal constituent space, suggesting significant differences in their metabolite profiles. Noticed, components of the ‘Supreme’ and ‘Premium’ grades seem to be highly similar. The hierarchical clustering analysis (HCA) result (Fig. 5B) is consistent with the PCA results. Based on the results of PCA and HCA, Euclidean distances of 1000 were selected as the grouping criterion, indicating that the samples could be discriminated into four groups (Group1-4). A PLS-DA model was constructed to screen for differential components among the four sample groups (R^2^X=0.913, R^2^Y=0.998, Q^2^=0.992, Fig. 5C). In the PLS-DA models, the 200 times of permutation test results (Intercepts, R^2^ = (0.0, 0.276), Q^2^ = (0.0, -0.599), Fig. 5D) indicate that the models are not overfitting. A total of 102 differential components were identified by the PLS-DA model using a VIP threshold of > 1.2. Among them, 23 differential components (Fig. 5E) were identified. Differential metabolites among the four sample groups were focused on flavonoid components and amino acids. Specifically, the significant discrepancies in flavonoid components were Epicatechin-(4*β*→8)-epigallocatechin-3-*O*-gallate, and Cyanidin 3-*O*-*β*-D-(6-*p*-coumaroylgalactoside), which were the most abundant in Special Tea. Amino acid components, such as _L_-tyrosine, _L_-tryptophan, _L_-phenylalanine, _L_-lysine, and so on, screened as differential components, are generally higher in Supreme and Premium tea, but lowest in Grade II. The differences among non-volatile components indicate significant variations in the biochemical status of tender shoots across grades and stages, which may affect tea functionality and taste. Therefore, the evaluation of vascular system protection activities and sensory attributes was carried out subsequently.

### 3.4 Vascular system protection activities

**Fig. 6:**
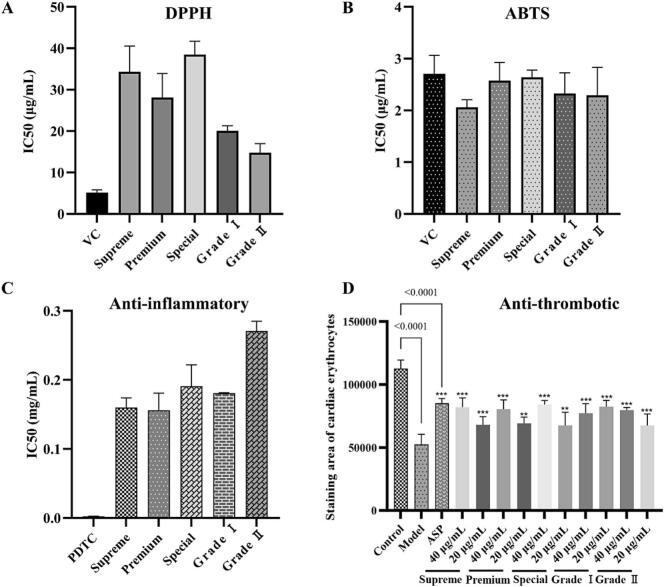
Vascular system protective activity of five grades of Duyun Maojian tea. (A) DPPH free radical-scavenging ability of Duyun Maojian tea. (B) ABTS free radical-scavenging ability of Duyun Maojian tea. (C) Results of anti-inflammatory activity in the LPS-induced inflammation model of RAW 264.7 cells; (D) Results of antithrombotic effects in the AH-induced zebrafish thrombosis model.

The antioxidant activity of different grades of Duyun Maojian was evaluated using the DPPH and ABTS methods. As demonstrated in Fig. 6A-B, five grades of Duyun Maojian samples showed comparative free radical scavenging capacity for both DPPH and ABTS (IC_50_ < 50 μg/mL). In particular, the free radical scavenging capacity of ABTS was comparable to that of the positive control (Vitamin C). These results suggest that the Duyun Maojian tea samples have potential antioxidant abilities. The Pearson correlation and PLS regression analyses were performed to investigate which compounds contributed to differences in antioxidant activity across tea grades, using DPPH IC_50_ values and the abundance of non-volatile components. 52 antioxidant-related compounds were obtained by Pearson correlation analysis using P < 0.05 and r < - 0.5 screening criteria. The PLS regression model was performed using screening thresholds of r < 0 and VIP > 1, yielding 192 activity-related constituents whose contents varied widely across samples. Finally, 47 intersect components were acquired, which were mainly phenolics and glycosides, including coniferoside, *c*-veratroylglycol, and 4-guanidinobutanoic acid. (Fig. S1A). In addition, the r and VIP values for the 47 components were presented in the SI. Table1.

The LPS-induced RAW 264.7 cells inflammation model was applied to assess the anti-inflammatory effects of the five tea samples. Cell viability of the tea extracts was also evaluated using the CCK-8 assay. Cell survival at the administered concentrations exceeded 90% for all extracts, indicating that the tea samples were not toxic to RAW 264.7 cells (Fig. S2). The anti-inflammatory activity of the extracts from the five tea samples showed a trend with quality grade (IC_50_ ranged from 0.15 to 0.30 mg/mL; Fig. 6C). In other words, Supreme and Premium tea have better anti-inflammatory activity than Grade II tea (P <0.05). The differential anti-inflammatory activity components were explored using Pearson's correlation and PLS regression analyses. As shown in Fig. S1B, 41 anti-inflammatory-related components were identified using Pearson correlation analysis. Then, 157 activity-related components were identified using PLS regression analysis. To improve the reliability of features related to anti-inflammatory activity, 41 intersecting components were identified, mainly alkaloids and catechins, including adenine, quinaldine, 3-methylindole, and (-)-epigallocatechin gallate (Ohishi et al., 2016)(SI. Table 2).

The AA-induced zebrafish thrombus model was used to evaluate the antithrombotic efficacy of 5 classes of tea samples. As shown in Fig. 6D, all five tea grades inhibited AA-induced zebrafish caudal vein thrombosis at 40 and 20 μg/mL. In addition, the different grades of tea at different test concentrations were significantly different from the model group (P < 0.05).

In summary, vascular disease is often accompanied by oxidative stress and inflammation, and thrombosis is the leading cause of death in clinical diseases. Based on the results of antioxidant, anti-inflammatory, and antithrombotic activities of the five different grades of tea, Duyun Maojian showed vascular protection effects, consistent with those reported for green tea functions. (Eshraghi et al., 2025; Quezada-Fernández et al., 2019; Stangl et al., 2006).

### 3.5 Youth consumer sensory evaluation

**Fig. 7:**
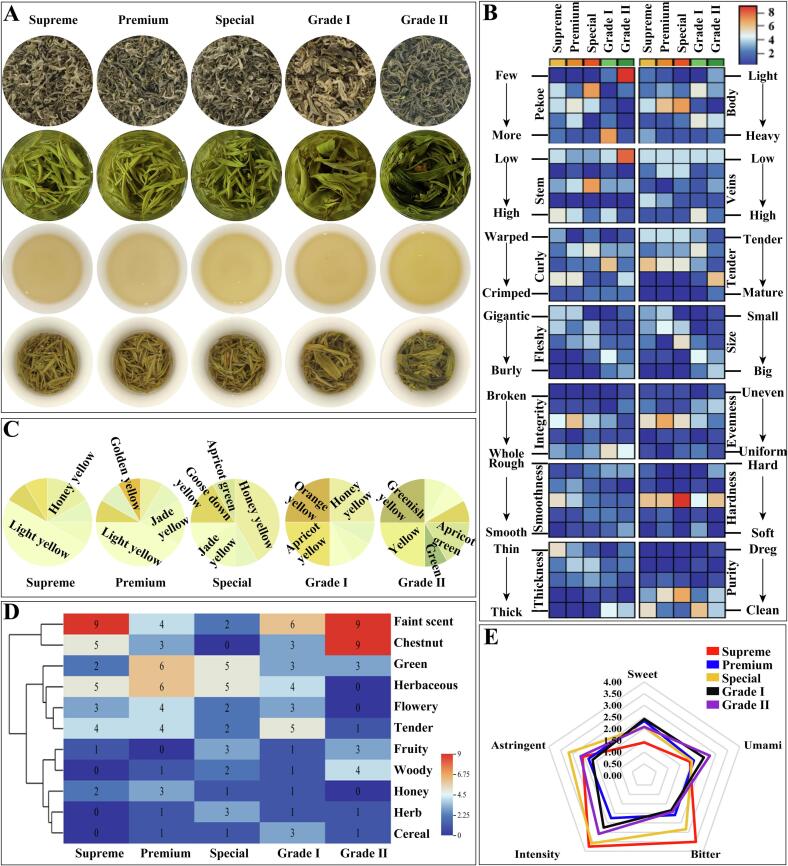
Results of sensory evaluation scores of five different grades of Duyun Maojian. (A) The appearance of dried tea, tea soup, infusion, and brewed tea leaves. (B) Heatmap of appearance assessment for dried tea leaves across five grades of Duyun Maojian. (C) Pie charts of the color recognition of tea soup for five different grades of Duyun Maojian. (D) Heatmap of aroma assessment for five grades of Duyun Maojian. (E) Radar plot of taste scores in five different grades of Duyun Maojian. A 5-point scale was used to evaluate all tea characteristics. For plots B, C, and D, the percentage or number of votes for each score is described. Plot E is denoted as the mean value of different degree scores of five different grades of Duyun Maojian.

Duyun Maojian grades were classified according to local standards (DB52/T 433-2018); however, whether consumers can accurately identify these levels remains to be seen. Thus, quantitative descriptive analysis (QDA) was used to describe its overall sensory characteristics from a consumer’s perspective. The organoleptic quality characteristics included the physical characteristics of the dried tea leaves, the color, aroma, and taste of the tea infusion, and the morphological characteristics of brewed tea leaves (Fig. 7A). The physical characteristics of the dried tea leaves are the first step in assessing the overall quality of the tea. According to the national sensory review standards (GB/T 23776–2018), the appearance characteristics of dried tea leaves were fully described using standardized terminology, and the results are shown in Fig. 7B. 14 attributes were assessed by scoring on a 1-5 scale. Each attribute matrix represents the number of scorers for that score. For the 'pekoe' attribute, most members marked ‘Grade I’ tea with most pekoe, and marked 'Grade II' teas with less pekoe. For the 'body' attribute, 'Grade II' and 'Grade I' were considered relatively dense, and 'Supreme' and 'Premium' grades were relatively light. Similarly, the review panel’s opinions on each attribute are mostly inconsistent, including 'Stem', 'Veins', 'Tender', 'Integrity', 'Evenness', 'Hardness', and 'Purity'. A relatively consistent rating opinion appears on 'Curly', 'Fleshy', 'Size', and 'Thickness' attributes. The evaluation results for appearance indicated that consumers cannot accurately distinguish 'Supreme' and 'Premium' grades, but can distinguish high-grade tea leaves from 'Grade II' and 'Grade I' teas.

The second step is to assess the tea soup's color, aroma, and taste. It is also the most important and most representative step of tea evaluation. The color of the tea broth from the five grades of Duyun Maojian is mainly yellowish, and it gradually deepens as the grade decreases (Fig. 7A). Among them, the 'Supreme' and 'Premium' grades were considered as light yellow mostly, and the special grade was primarily honey yellow. There are significant differences in color recognition between 'Grade II' and 'Grade I' (Fig. 7C), which can be attributed to assessors being influenced by the background color of the bottom leaves, which align with common people’s brewing and drinking habits.

Smell is a vital indicator of tea quality. The most characteristic faint scent aroma is present in five grades of Duyun Maojian (J. Yu et al., 2024). As shown in Fig. 7D, the 'Supreme' was mainly assessed to have faint, chestnut, and herbaceous aroma characteristics. The 'Premium' and 'Special' grades were assessed for prominent herbaceous and green aroma characteristics. In addition, the 'Grade I' also showed a tender aroma, and the ‘Grade II’ had a chestnut and woody aroma. These results indicated that the reviewers could distinguish olfactory differences between 'Supreme' and 'Premium' and that the olfactory characteristics of the five tea types differed significantly.

In addition to aroma, taste is also a significant characteristic for judging the sensory quality of tea. Five characteristic attributes were selected as evaluation indices, including bitterness, astringency, umami, sweetness, and concentration intensity, to evaluate the flavor characteristics of green tea. Fig. 7E shows the scores for the flavor profile. The five grades of tea samples had lower sweetness (scores < 2.5) and higher bitterness, astringency, and concentration. The bitterness and astringency in green tea are mainly attributed to polyphenolic and alkaloid components (Deng et al., 2022). Polyphenols are the significant components in green tea, including catechins, small-molecule phenolic acids, tannins, and flavonoid glycosides. Catechins are the principal constituents of tea polyphenols in green tea, especially (−)-epigallocatechin gallate (EGCG), (−)-epigallocatechin (EGC), (−)-epigallocatechin gallate (ECG), and (−)-epigallocatechin (EC). Among these compounds, ECG had the highest bitter and astringent flavor (Xu et al., 2018). Quercetin-3-*O*-rutinoside and quercetin-3-*O*-galactoside were the primary flavonoid glycosides (P. Yu et al., 2014). Alkaloids, including caffeine, theanine, and theobromine, also contributed to the bitter flavor. The combined effect of the above components endowed bitter and astringent sensory properties (L. Zhang et al., 2020).

**Fig. 8:**
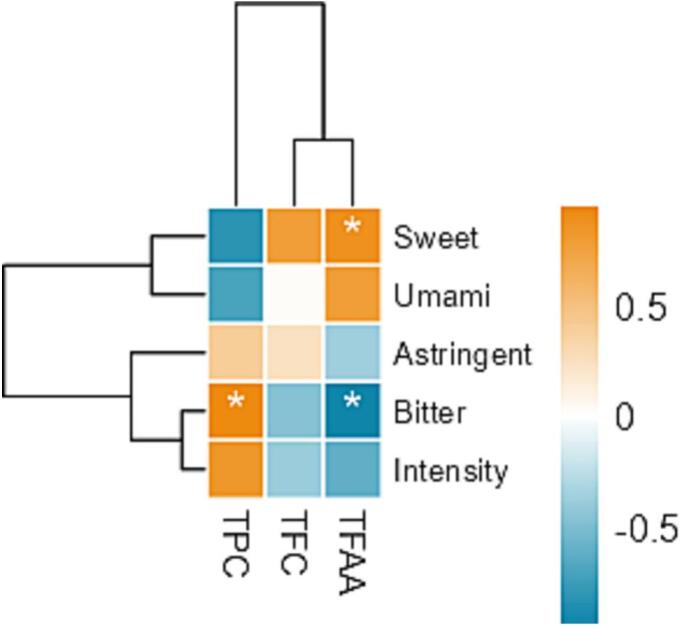
Heatmap of Pearson's correlation coefficients between TPC, TFC, TFAA, and taste scores. Asterisks indicate statistically significant correlations (P < 0.05).

Pearson correlation analysis further indicated that TPC content was positively correlated with both bitterness and astringency (r > 0.5, P < 0.05), suggesting that tea polyphenols are the main components driving the bitter and astringent tastes of Duyun Maojian tea. Among the five grades of tea, the 'Supreme' had the highest bitterness and intensity of concentration, which was consistent with the results of the TPC measurements and Pearson correlation analysis (Fig. 1 and Fig. 8). Previous studies have shown that amino acids are the main substances that affect the umami of tea (L. Zhang et al., 2020). The taste evaluation results suggest that TFAA shows trends affecting the umami flavor of Duyun Maojian (Fig. 7E), despite its lower amino acid content. As shown in Fig. 8, Pearson correlation analysis revealed a positive correlation between TFAA content and umami taste (r > 0.5), indicating that the intensity of umami perception in Duyun Maojian tea is closely related to its amino acid components. Based on the supporting literature above and Pearson correlation analysis, it can be concluded that the bitter and astringent tastes in Duyun Maojian tea are primarily influenced by polyphenolic compounds, whereas the umami perception is mainly driven by amino acids.

The brewed tea leaves are an important part of the tea's overall quality. To have a more comprehensive understanding of the sensory quality of Duyun Maojian, 12 characteristic attributes associated with brewed tea leaves were depicted (Fig. 7A, Fig. S2), including 'stem', 'veins', 'unfold', 'tenderness', 'gigantic and burly', 'big and small', 'integrity', 'evenness', 'smoothness', 'hardness', 'thickness', and 'purity'. The evaluation results unanimously indicated that the high-grade teas ('Supreme', 'Premium', and 'Special') in terms of integrity, evenness, smoothness, and purity surpass those of the low-grade teas ('Grade I' and 'Grade II'). At the same time, it is also believed that high-grade teas are more tender than low-grade teas. Regarding size, the lower quality leaves appeared to have a larger, more spreading profile. In summary, the sensory descriptions of the dried and brewed tea leaves aligned with the provincial standard (DB52/T 433-2018) for the grade classification of Duyun Maojian, offering unique insights into its different grades from a non-professional perspective.

## 4 Conclusion

As a national geographical indication product, Duyun Maojian has not yet received sufficient reports on its ingredients, activity, and sensory evaluation. Our study evaluates the quality characteristics of five grades of Duyun Maojian, including determination of TPC, TFC, and TFAA; untargeted metabolomics analysis (LC-MS/MS and GC-MS); health-related vascular-protection activity; and consumer sensory analysis. Determination of TFAA showed Grade I (40775.76 μg/g) and Grade II (36263.64 μg/g) exhibited the highest TFAA levels with no significant difference between them. 'Supreme' possessed the highest TPC, reaching 21936.86 μg GAE/g sample. GC-MS detected 284 aroma compounds, including 78 grade-discriminative volatiles with terpenes, alkanes, and esters contributing to the differences. LC-MS/MS identified 23 key differential metabolites, with flavonoid components and amino acids serving as core markers for discrimination between sample groups. Vascular system protective activity was demonstrated through antioxidant, anti-inflammatory, and antithrombotic evaluations of Duyun Maojian. ABTS scavenging activity of Duyun Maojian extracts was higher than that of vitamin C. The anti-inflammatory effects show a trend correlated with grade. The extracts exhibit anti-thrombotic activity at 20 μg/mL. Finally, sensory profiles from consumers’ perspectives revealed grade-dependent bitterness (polyphenol-driven) and umami (amino acid-associated) flavor. These results provide system-level insights into Duyun Maojian, serving as a foundation for further resource development and utilization.

## CRediT authorship contribution statement

**Wei-wu Xia:** Writing – original draft, Visualization, Validation, Investigation, Formal analysis. **Ting Li:** Validation, Software, Data curation. **Hui-min Fang:** Visualization. **Fei-bi Xiong:** Visualization. **Lu-lu Deng:** Writing – review & editing, Funding acquisition. **Jiang Li:** Writing – review & editing. **Xiao-jiang Hao:** Writing – review & editing, Supervision. **Peng Zhang:** Writing – review & editing, Funding acquisition, Data curation. **Shu-zhen Mu:** Writing – review & editing, Supervision, Project administration, Funding acquisition, Conceptualization.

## Ethical statement

All zebrafish experiments were approved by the Animal Care and Welfare Committee of Guizhou Medical University (No. 2502559) and were conducted in accordance with relevant national regulations and internationally recognized guidelines, including the NIH Guide for the Care and Use of Laboratory Animals.

For the sensory evaluation, given that all samples used in the sensory evaluation were non-toxic and without adverse effects; therefore, formal ethical approval was not required by our institution. Nevertheless, appropriate protocols were implemented to safeguard the rights and privacy of all participants. Each panelist was fully informed about the purpose and procedures of the study and took part voluntarily. Informed consent was obtained from every participant before the sensory evaluation, and all data were anonymized to ensure confidentiality. Participants were also informed of their right to withdraw from the study at any time without consequence.

## Declaration of competing interest

The authors declare that they have no known competing financial interests or personal relationships that could have appeared to influence the work reported in this paper.

## Data Availability

Data will be made available on request.
