# Supplementary material for: Different grades of green tea ‘Duyun Maojian’: a comprehensive constituent, bioactivity, and sensory evaluation from the consumer’s specific perspective
Source: Food Chem X. 2026 Apr 7;35:103843. doi: 10.1016/j.fochx.2026.103843 (PMC13091367; doi:10.1016/j.fochx.2026.103843)
Supplement: Supplementary material [file mmc1.docx]

**Different Grades of Green Tea 'Duyun Maojian': A Comprehensive Constituent, Bioactivity, and Sensory Evaluation from the Consumer's Specific Perspective**

Wei-wu **Xia^a^**, Ting **Li^a^**, Hui-min **Fang****^b^**, Fei-bi **Xiong^c^**, Lu-lu **Deng^a^**, Jiang **Li^a^**, Xiao-jiang **Hao^a,^ ^d, *^**, Peng **Zhang^a, *^**, Shu-zhen **Mu^a *^**

**^a^** State Key Laboratory of Discovery and Utilization of Functional Components in Traditional Chinese Medicine, Natural Products Research Center of Guizhou Province, School of Pharmaceutical Sciences, Guizhou Medical University, Guiyang, 550014, China

**^b^** College of Pharmacy, Guizhou University, South Section of Huaxi Road 2078, Guiyang 550025, China

**^c^** College of Pharmacy, Guizhou University of Traditional Chinese Medicine, Huaxi University Town, Guiyang, 550025, China

**^d^** Kunming Institute of Botany, Chinese Academy of Sciences, 132 Lanhei Road, Kunming 650201, China

**Supplementary Information**

**Table of contents**

**Fig. S1.** (A and B) The intersection components obtained by Pearson correlation analysis and PLS regression modeling for antioxidant and anti-inflammatory, respectively.

**Fig. S2.** The cellular viability of the anti-inflammatory assay in five teas, respectively.

**Fig. S3.** Results of sensory evaluation scores of the brewed tea leaves for five different grades of Duyun Maojian. (A) Bar charts of 12 characteristics of brewed tea leaves, portrayed by the percentage of people under each score. (B) Radar plot of the 12 features of the brewed tea leaves, depicted by selecting the feature scores with the highest percentage of people.

**Table 1.** The antioxidant activity-related differential metabolites (r < 0; VIP > 1; P < 0.05).

**Table 2.** The anti-inflammatory activity-related differential metabolites (r < 0; VIP > 1; P < 0.05).

**Section 1.** Major method and instrument parameters for volatile components detection by GC-MS

**Section 2.** Major Liquid Chromatography–Mass Spectrometry (LC-MS) Conditions for non-volatile components detection

**Section 3.** Parameters used in the Mzmine workflow for LC-MS data processing.

**Section 4.** R code for LC-MS data pre-processing


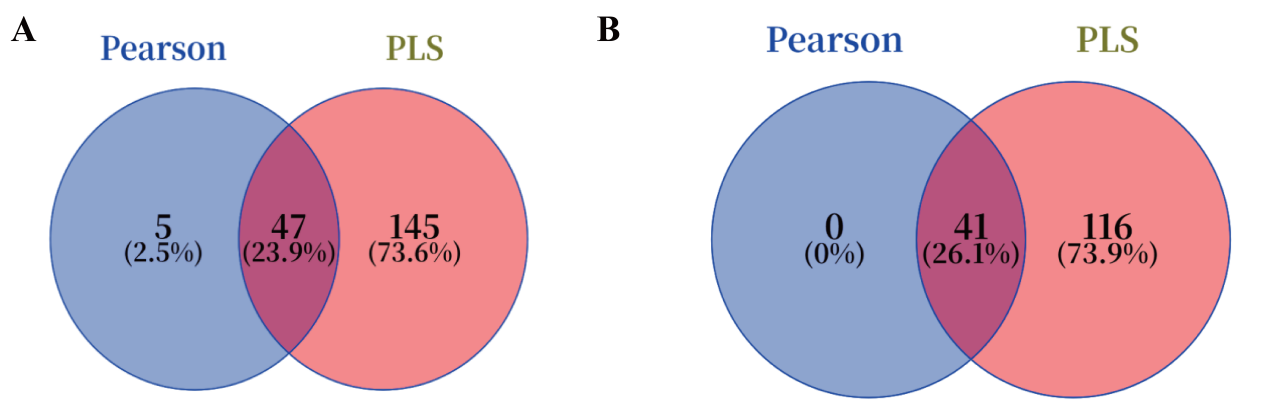


**Fig. S1.** (A and B) The intersection components obtained by Pearson correlation analysis and PLS regression modeling for antioxidant and anti-inflammatory, respectively.


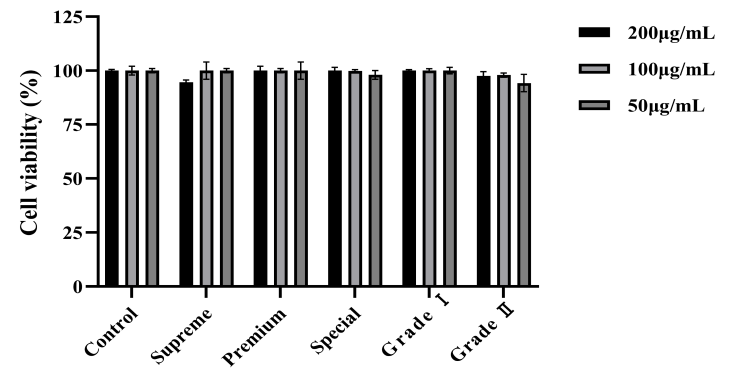


**Fig. S2.** The cellular viability of the anti-inflammatory assay in five teas, respectively.


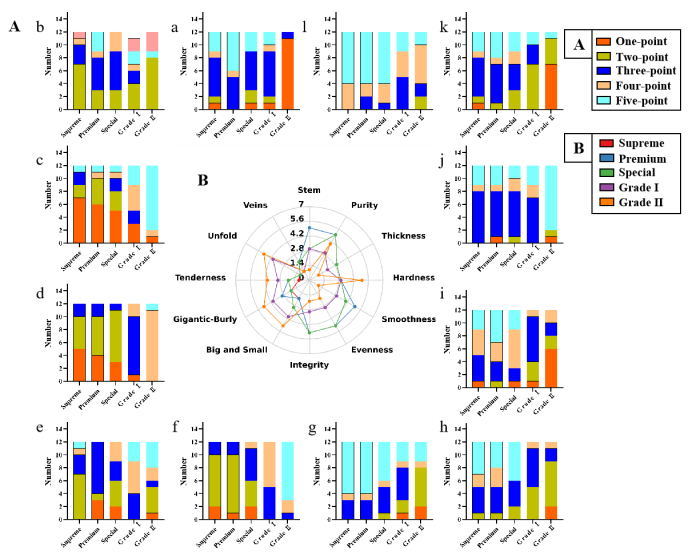


**Fig. S3**. Results of sensory evaluation scores of the brewed tea leaves for five different grades of Duyun Maojian. (A) Bar charts of 12 characteristics of brewed tea leaves, portrayed by the percentage of people under each score. (B) Radar plot of the 12 features of the brewed tea leaves, depicted by selecting the feature scores with the highest percentage of people.

**Table 1.** The antioxidant activity-related differential metabolites (r < 0; VIP > 1; P < 0.05).

| NO. | ID | Compound name | r | VIP | P |
| --- | --- | --- | --- | --- | --- |
| 1 | 1020 | - | -0.876092459 | 2.10621 | 1.84904E-05 |
| 2 | 4793 | - | -0.813769826 | 1.96833 | 0.000223061 |
| 3 | 1022 | - | -0.80652092 | 2.06972 | 0.000280615 |
| 4 | 1152 | - | -0.793455738 | 2.15237 | 0.000414856 |
| 5 | 1031 | - | -0.780008481 | 2.10605 | 0.000603613 |
| 6 | 3025 | - | -0.766823364 | 1.82401 | 0.00085132 |
| 7 | 6637 | - | -0.73124443 | 1.88704 | 0.001950385 |
| 8 | 4667 | - | -0.73093512 | 1.72439 | 0.001963403 |
| 9 | 10445 | - | -0.722162985 | 1.82942 | 0.002362693 |
| 10 | 15128 | - | -0.706323551 | 1.79509 | 0.003246871 |
| 11 | 6551 | - | -0.699477981 | 1.68229 | 0.00370251 |
| 12 | 9081 | - | -0.695394817 | 1.46598 | 0.00399761 |
| 13 | 3826 | - | -0.689481082 | 1.79948 | 0.004457843 |
| 14 | 13341 | - | -0.687450012 | 2.01576 | 0.004625258 |
| 15 | 12632 | - | -0.669748259 | 1.52816 | 0.006305313 |
| 16 | 9730 | - | -0.662387783 | 1.60232 | 0.007131394 |
| 17 | 14736 | - | -0.659135567 | 1.77019 | 0.007522448 |
| 18 | 14856 | - | -0.656526288 | 1.50423 | 0.007848208 |
| 19 | 5784 | - | -0.651359018 | 1.69236 | 0.008525942 |
| 20 | 12634 | - | -0.645768747 | 1.46788 | 0.009309909 |
| 21 | 2214 | 4-GUANIDINOBUTANOATE | -0.643689706 | 1.48316 | 0.009615426 |
| 22 | 1864 | - | -0.633328632 | 1.5068 | 0.011256636 |
| 23 | 1139 | - | -0.621422507 | 1.41185 | 0.013403175 |
| 24 | 4950 | - | -0.609374849 | 1.60604 | 0.015885034 |
| 25 | 586 | - | -0.609100347 | 1.38192 | 0.015945435 |
| 26 | 3341 | - | -0.608262003 | 1.39954 | 0.016130999 |
| 27 | 7180 | - | -0.600148731 | 1.38233 | 0.018014061 |
| 28 | 13031 | - | -0.59934637 | 1.27749 | 0.018209072 |
| 29 | 8606 | - | -0.599014533 | 1.62426 | 0.018290196 |
| 30 | 1837 | - | -0.588813726 | 1.48877 | 0.020922178 |
| 31 | 10889 | Coniferoside | -0.587699845 | 1.32704 | 0.021226209 |
| 32 | 2086 | - | -0.57174917 | 1.36079 | 0.02596048 |
| 33 | 1091 | (2,3-dihydroxyl-1-(4-hydroxy-3-methoxyphenyl)-propan-1-one | -0.571435471 | 1.28022 | 0.02606098 |
| 34 | 2477 | - | -0.560939299 | 1.34274 | 0.029596931 |
| 35 | 11453 | - | -0.559079865 | 1.34948 | 0.030259284 |
| 36 | 10732 | - | -0.557215816 | 1.43277 | 0.030934433 |
| 37 | 943 | Arginine\|2-amino-5-(diaminomethylideneamino)pentanoic acid | -0.555689867 | 1.29572 | 0.031495516 |
| 38 | 6366 | - | -0.551939997 | 1.13855 | 0.032906834 |
| 39 | 6983 | (2S)-1-(3',4',5'-trihydroxyphenyl)-3-(2'',4 '',6''-trihydroxyphenyl)-propan-2-ol | -0.550064461 | 1.22789 | 0.033630283 |
| 40 | 6791 | - | -0.543744732 | 1.3907 | 0.036156038 |
| 41 | 6349 | - | -0.524490195 | 1.37847 | 0.044728617 |
| 42 | 15055 | - | -0.523236017 | 1.18064 | 0.045334873 |
| 43 | 15222 | - | -0.523096203 | 1.39362 | 0.045402832 |
| 44 | 13915 | - | -0.521370772 | 1.41991 | 0.046247724 |
| 45 | 13803 | - | -0.521260483 | 1.2783 | 0.046302121 |
| 46 | 13242 | - | -0.52108848 | 1.17949 | 0.046387051 |
| 47 | 7141 | - | -0.518354424 | 1.2453 | 0.047752561 |

Note:“-” indicates unannotated results

**Table 2.** The anti-inflammatory activity-related differential metabolites (r < 0; VIP > 1; P < 0.05).

| NO. | ID | Compound name | r | VIP | P |
| --- | --- | --- | --- | --- | --- |
| 1 | 6417 | 3-Indoleacrylic acid | -0.745529324 | 1.7213 | 0.001420414 |
| 2 | 10911 |  | -0.7452434 | 1.78219 | 0.001429738 |
| 3 | 6435 | L-Tryptophan | -0.742320494 | 1.70752 | 0.001527909 |
| 4 | 6424 | Benzyl nitrile/'Indole | -0.741810091 | 1.6932 | 0.001545595 |
| 5 | 6419 | 3-Methylindole | -0.735337465 | 1.72798 | 0.001784547 |
| 6 | 865 | L-Lysine | -0.73356602 | 1.68127 | 0.001854869 |
| 7 | 6428 | Abrine | -0.732635799 | 1.67382 | 0.001892678 |
| 8 | 6415 | 2-Methylquinoline/' 7-Methylquinoline | -0.728655142 | 1.69715 | 0.002061533 |
| 9 | 6406 | - | -0.726995515 | 1.67656 | 0.002135409 |
| 10 | 6413 | - | -0.726354322 | 1.68991 | 0.002164513 |
| 11 | 6433 | - | -0.72535573 | 1.66735 | 0.002210472 |
| 12 | 6439 | Hexadecane | -0.716197714 | 1.65456 | 0.002669556 |
| 13 | 6421 | - | -0.687212944 | 1.58226 | 0.004645119 |
| 14 | 2413 | L-Tyrosine | -0.681932218 | 1.58266 | 0.005105349 |
| 15 | 877 | Pipecolic acid. Hexahydropicolinic acid. Pipecolinic acid. Homoproline/'N-ethyl-5-hydroxy-2-pyrrolidinone | -0.681297393 | 1.57261 | 0.00516302 |
| 16 | 10145 | Epicatechin(4belta-8)epigallocatechin 3-O-gallate/'Epigallocatechin(4belta-8)epicatechin 3-O-gallate/'(+)-Catechin (4alfa-8)-(-)-epigallocatechin 3-O-gallate | -0.675933922 | 1.84347 | 0.005671125 |
| 17 | 10146 | - | -0.675591224 | 2.41371 | 0.005704884 |
| 18 | 9106 | - | -0.66152253 | 1.51179 | 0.007233836 |
| 19 | 10918 | - | -0.658225933 | 1.81587 | 0.007634783 |
| 20 | 6431 | - | -0.653338644 | 1.489 | 0.008261087 |
| 21 | 2005 | - | -0.642702792 | 1.5368 | 0.009763167 |
| 22 | 6014 | Adenine， Vitamin B4 | -0.638438029 | 1.43236 | 0.010422103 |
| 23 | 4815 | - | -0.638211701 | 1.45628 | 0.010458017 |
| 24 | 4820 | - | -0.634890926 | 1.43528 | 0.010996134 |
| 25 | 4798 | - | -0.634112093 | 1.43274 | 0.011125405 |
| 26 | 4809 | L-Phenylalanine\|(2S)-2-Azaniumyl-3-phenylpropanoate | -0.633257206 | 1.4387 | 0.011268659 |
| 27 | 4832 | - | -0.631440366 | 1.42591 | 0.011577873 |
| 28 | 4824 | - | -0.630945893 | 1.41051 | 0.011663161 |
| 29 | 3311 | L-Tyrosine | -0.630613814 | 1.4515 | 0.011720712 |
| 30 | 11767 | EPIGALLOCATECHIN GALLATE, (-) | -0.628432076 | 1.3711 | 0.012104329 |
| 31 | 4807 | - | -0.626883123 | 1.42186 | 0.012382551 |
| 32 | 7404 | - | -0.616159728 | 1.08381 | 0.014447343 |
| 33 | 4823 | Benzyl nitrile/'Indole/Indole | -0.61496394 | 1.35738 | 0.014693125 |
| 34 | 6012 | MeSAdo | -0.611665621 | 1.39813 | 0.015387826 |
| 35 | 4821 | - | -0.611218147 | 1.30647 | 0.015483992 |
| 36 | 13074 | - | -0.595118027 | 1.64504 | 0.019263667 |
| 37 | 8607 | Salicylaldehyde/'Benzoic Acid, Benzoate | -0.590735298 | 1.26296 | 0.020405525 |
| 38 | 13347 | - | -0.587233692 | 1.54417 | 0.021354441 |
| 39 | 11753 | - | -0.561692369 | 1.2712 | 0.029331813 |
| 40 | 3497 | di-ACR-TP | -0.552894344 | 1.5023 | 0.032543233 |
| 41 | 4303 | - | -0.548652056 | 1.29476 | 0.034182916 |

Note: “-” indicates unannotated results

**Section 1.** Major method and instrument parameters for volatile components detection by GC-MS

**Gas Chromatography (GC) Conditions:**

**Column:** Agilent HP-5ms Ultra Inert (30 m × 250 μm × 0.25 μm)

**Inlet:** Splitless mode, temperature 250°C, pressure 7.07 psi

**Carrier gas:** Helium, constant flow mode at 1.0 mL/min

**Oven temperature program:**

**Initial:** 40°C held for 2 min

**Ramp 1:** 4°C/min to 140°C (no hold)

**Ramp 2:** 2°C/min to 160°C, held for 5 min

**Ramp 3:** 10°C/min to 310°C (no hold)

**Total run time:** 57 min, followed by a 2 min post-run at 310°C

**Injection volume:** Manual injection

**Transfer line temperature:** 250°C

**Mass Spectrometry (MS) Conditions:**

**Ion** **source:** EI source

**Collision gas:** Nitrogen at 1.5 mL/min

**Quench gas:** Helium at 2.25 mL/min

**Data acquisition:** Multiple signals recorded at 50 Hz (labeled as test spectra)

**Collision cell pressure:** 10 psi (constant)

**Section 2.** Major Liquid Chromatography–Mass Spectrometry (LC MS) Conditions for non-volatile components detection

**Ion Source Conditions:**

**Ionization mode:** Positive and negative polarity (method set to positive polarity)

**Spray voltage:** +3400 V (positive), –3100 V (negative)

**Capillary temperature:** 350 °C (positive), 320 °C (negative)

**Sheath gas flow:** 45 arb (positive), 40 arb (negative)

**Auxiliary gas flow:** 15 arb (positive), 10 arb (negative)

**Probe heater temperature:** 350 °C

**S-lens RF level:** 60 %

**Mass Spectrometer Settings:**

**Mass analyzer:** Orbitrap

**Full‑MS resolution:** 70,000 (at m/z 200)

**Scan range:** m/z 100–1500

**Automatic gain control (AGC) target:** 1×10⁶

**Maximum injection time:** Auto

**Data‑dependent MS² (dd‑MS²) resolution:** 17,500

**Isolation window:** 1.0 m/z

**Normalized collision energy (NCE):** Stepped 30, 40, 60 %

**AGC target for MS²:** 5×10⁴

**Dynamic exclusion:** Enabled (auto settings)

**Liquid Chromatography (Partial):**

**Method duration:** 21.00 min

**Valve switching:** Divert valve switched to waste position at 20.21 min

**Syringe pump (if used):** Hamilton syringe, 3.0 µL/min flow rate (250 µL volume)

**Section 3.** Parameters used in the Mzmine workflow for LC-MS data processing.

Raw LC–MS data were processed using MZmine 4.3.0 to extract, align, and annotate metabolic features. The batch workflow comprised the following sequential modules:

**1. Data Import**

Files: 32 mzML files (11 blanks, 15 samples in triplicate, 5 quality controls) from different grades of Duyun Maojian tea.

Scan filters: All MS levels, any polarity, spectrum type ANY.

**2. Mass Detection**

MS¹ scans: Retention time (RT) range 0–12 min; mass detection algorithm Centroid with noise level = 10,000.

MS² scans: RT range 0–12 min; Centroid detection with noise level = 0.

**3. Chromatogram Building**

Module: ADAP Chromatogram Builder.

Minimum consecutive scans: 5.

Minimum intensity for consecutive scans: 30,000.

Minimum absolute height: 70,000.

m/z tolerance (scan-to-scan): 0.02 (absolute).

**4. Chromatographic Deconvolution**

Module: Minimum Search Feature Resolver.

Chromatographic threshold: 0.9.

Minimum absolute height: 70,000.

Min ratio of peak top/edge: 1.7.

Peak duration range: 0–1 min.

Minimum scans per peak: 5.

MS¹–MS² pairing tolerance: m/z 0.02 (absolute), RT tolerance 0.1 min.

**5. Isotope Grouping**

m/z tolerance: 0.02 (absolute).

RT tolerance: 0.1 min.

Maximum charge: 1.

Representative isotope: Most intense.

Monotonic shape: Enabled.

Never remove feature with MS²: True.

**6. Feature Alignment (Join Aligner)**

m/z tolerance (sample-to-sample): 0.02 (absolute), weight = 3.0.

RT tolerance: 0.1 min, weight = 1.0.

Require same charge state: True.

Isotope pattern and spectral similarity comparisons: Not used in this alignment.

**7. Feature Filtering**

Retention time range kept: 0–12 min.

Require feature to have MS² scan: True.

Keep rows that match all criteria.

Never remove feature with MS²: True.

**Key Processing Parameters Summary:**

| **Step** | **Main Algorithm/ Criteria** | **Critical Settings** |
| --- | --- | --- |
| **Mass Detection** | Centroid (MS¹ & MS²) | Noise level: 10,000 (MS¹), 0 (MS²) |
| **Chromatogram Building** | ADAP | Min consecutive scans: 5; Min intensity: 30,000; Min height: 70,000 |
| **Deconvolution** | Minimum Search Resolver | Min height: 70,000; Peak top/edge ratio: 1.7; Min scans: 5 |
| **Isotope Grouping** | Isotope Grouper | m/z tol.: 0.02; RT tol.: 0.1 min; Max charge: 1 |
| **Alignment** | Join Aligner | m/z tol.: 0.02 (weight 3); RT tol.: 0.1 min (weight 1) |
| **Filtering** | Rows Filter | RT range: 0–12 min;Keep only features with MS² |

**Section 4.** R code for LC-MS data pre-processing

##LC-MS data pre-processing

DY_TEA_LC <- read.csv("DY-TEA-LC.csv")

MS <- data.frame(DY_TEA_LC[, c(1:3)], DY_TEA_LC[,c(4:23)]-1000)

MS_subB1000 <- data.frame(MS[, c(1:3)], replace(MS[, c(4:23)], MS[, c(4:23)] < 0, 0))

MS_subB1000_de0 <- MS_subB1000[which(rowSums(MS_subB1000[, -c(1:3)]) > 0),]

QC <- MS_subB1000_de0[, c(19:23)]

MS_subB1000_de0_QC0 <- MS_subB1000_de0[rowMeans(QC==0) < 0.1, ]

data1 <- MS_subB1000_de0_QC0

write.csv(data1, "data1.csv")

sample.info <- read.csv("sample.info.csv")

remotes::install_github("jaspershen/MetNormalizer")

library(MetNormalizer)

metNor(

ms1.data.name = "data.csv",

sample.info.name = "sample.info.csv",

minfrac.qc = 0,

minfrac.sample = 0,

optimization = TRUE,

multiple = 5,

threads = 4

)
